# Computationally Efficient Implementation of Joint Detection and Parameters Estimation of Signals with Dispersive Distortions on a GPU

**DOI:** 10.3390/s22093105

**Published:** 2022-04-19

**Authors:** Vladislav I. Lipatkin, Evgeniy M. Lobov, Nikolai A. Kandaurov

**Affiliations:** Science and Research Department, Moscow Technical University of Communications and Informatics, Moscow 111024, Russia; lobovrts@yandex.ru (E.M.L.); kandaurov@srd.mtuci.ru (N.A.K.)

**Keywords:** DSP, GPU, FFT, communications, dispersion distortions, Doppler shift, ionosphere, radar

## Abstract

The detector is an integral part of the device for receiving and processing radio signals. Signals that have passed through the ionospheric channel acquire an unknown Doppler shift and are subject to dispersion distortions. It is necessary to carry out joint detection and parameter estimation to improve reception quality and detection accuracy. Modern hardware base developing makes it possible to implement a device for joint detection and evaluation of signals based on standard processors (CPU) and graphic processors (GPU). The article discusses the implementation of a signal detector that allows for real-time operation. A comparison of implementations of algorithms for estimating the Doppler frequency shift through multiplication by a complex exponent and the fast Fourier transform (FFT) is performed. A comparison of computational costs and execution speed on the CPU and GPU is considered.

## 1. Introduction

Ionospheric radio communication is a highly reliable and cost-effective solution for organizing communication with outlying regions, as well as with regions whose infrastructure has been disrupted due to natural disasters. Currently, development of decameter ionospheric radio communication systems is on the way to increasing the speed of information transmission [1,2,3,4,5,6]. When using broadband signals in the decameter range, the frequency dispersion has a significant effect on the signal [7,8,9,10,11,12,13,14]. Thus, due to the frequency dispersion, at different frequencies wideband signals have different propagation delays. Such a difference leads to a synchronization error and affects the quality of signal detection and the quality of information reception [15,16,17]. A separate problem is the detection of long signal preambles with a duration of about several seconds long, with a spectrum wider than 100 kHz [18,19,20] and with a coherent accumulation of the detected signal energy throughout its duration. In this case, the signal base reaches a value exceeding 50 dB, and the required accuracy of estimation and compensation of the Doppler frequency shift is in the tenths and in some cases hundredths of a hertz. Otherwise, the coherent accumulation of signal energy over time intervals of units or even tens of seconds becomes impossible. Simultaneously, with the evaluation of the Doppler shift [21,22,23,24,25,26], it is also required to evaluate and compensate for the dispersion distortions of the detected signals.

In this paper, we show the possibility of constructing a device for the joint detection and estimation of the parameters of signals with dispersion distortions on graphic processors. Implementations proposed in this paper allow for the simultaneous detection of signals and estimation of dispersion distortions, delay, Doppler shift, and initial phase in real time.

## 2. Related Work

Stein, Tolimieri, and Winograd are the founders of research on algorithms for calculating uncertainty functions. Stein has described a processing approach for obtaining joint delay and frequency offset (DTO/DFO) estimates for continuous signals based on the efficient calculation of complex ambiguity functions [27]. Typically, it involves a two-mode process called coarse and fine modes. Coarse mode is used to greatly reduce the time delay and frequency offset uncertainty, after which fine mode calculations are performed. Precise mode uses product/filter mixing interpretation, greatly reducing the processing load. Tolimieri and Winograd proposed an algorithm for the discrete ambiguity function calculation in [28]. They rely on the fact that, in most basic applications, it is necessary to calculate the limited parts of the DFT of a discrete ambiguity function. To do this, they first pass a long sequence through a decimated FIR filter, and then they use the FFT algorithm. Additionally, computationally efficient algorithms for the joint estimation of the Doppler shift and time delay are considered in [29,30]. These papers propose a new method based on a pre-weighted Zoom FFT with a cascaded filter algorithm to minimize the processing load of cross-ambiguity functions without compromising performance. The weighting process in the Zoom FFT method provided an opportunity for the researchers to get rid of redundant calculations. The multi-stage filtering method was used to reduce complexity and to obtain a high-performance system. A method for processing segments was also proposed, adapted to calculate the ambiguity function when imposing input data frames. By considering the calculation of the cross-ambiguity functions of overlapping data frames as the calculation of the FFT of the overlapping data, the redundancy of the calculations can be eliminated.

Modern techniques for reducing the complexity of the cross-ambiguity function (CAF) are based on numerical fitting for CAF [31]. These algorithms make full use of the property that the CAF is symmetrical in the frequency domain. Simulation results show that, compared to the method that looks for the CAF peak, the proposed algorithm can significantly reduce computational complexity while meeting the accuracy requirements of the joint time-frequency estimate.

In paper [32], the authors propose a method for solving the problem of determining the mutual delay time of ultra-wideband signals. A modified algorithm, which can be implemented by parallel calculation of the cross-ambiguity function, was used to compensate Doppler shift of the recorded signals. This algorithm was based on the division of an ultra-wideband signal into separate frequency channels. An increase in the computational efficiency of the proposed algorithm was achieved by parallel calculation of the convolution function and cross-ambiguity.

However, all the above works do not take into account the problem of compensating for dispersion distortions and processing signals with a base over 50 dB. There are also no computationally efficient solutions implemented on the GPU that allow for the real-time detection of signals with a base of more than 50 dB (the signal spectrum width is hundreds of kHz, the duration is a few seconds) with the simultaneous estimation of dispersion distortions, delay, Doppler shift, and initial phase. Given these features, the joint detection and estimation of signal parameters requires large computational resources. The modern technology level makes it possible to consider the possibility of developing a computationally efficient implementation of various algorithms on GPUs. For example, such GPU implementation allows you to build systems for parallel simulation of MIMO radars [33] and build digital downconverter [34]. Additionally, GPUs are very often used in deep learning [35]. Thus, computing on GPUs is becoming more and more efficient.

## 3. Analytical Formulation of the Problem

The complex envelope of the signal at the joint detection and signal parameters estimation device input can be represented as a composition of the useful signal complex envelope, distorted by the frequency dispersion of ionospheric channel, and the complex envelope of white Gaussian noise:

(1) $$\begin{array}{c} {\dot{y}}_{i}(\varphi ,\tau =l\cdot \Delta t,f_{d},s)=e^{-j\varphi }e^{j2\pi f_{d}\left(i-l\right)\Delta t}{\dot{x}}_{i-l}\left(s\right)+{\dot{n}}_{i},\\ i=0÷N_{p}-1, \end{array}$$

where $\dot{\bar{x}}\left(s\right)=\dot{\bar{x}}*\dot{\bar{h}}(s)$ is distorted by the ionospheric channel useful signal complex envelope, ${\dot{h}}_{i}\left(s\right)$ is the ionospheric channel impulse response (IR) complex envelope, ${\dot{x}}_{i}$ is the complex envelope of useful undistorted signal, $f_{d}$ is the doppler frequency shift, τ is the delay in seconds, l is the delay in samples, Δt is the sample time, s is the slope of the dispersion characteristic (parameter that characterizes dispersion distortions), φ is the unknown phase shift, $\dot{n}(t)$ is the complex envelope of white Gaussian noise with zero mean and variance $\sigma_{ɯ}^{2}$, and $N_{p}$ is the number of samples.

The ionospheric channel impulse response (IR) complex envelope connects with frequency response of the ionospheric channel $\dot{H}(j2\pi f)$ through Fourier transform $\dot{H}(j2\pi f)$:$\dot{h}\left(t,s\right)=\int_{-\infty }^{\infty }\dot{H}(j2\pi f)e^{j2\pi f}df$, where x(t) is a transmitted signal that is known at the receiving side.

The ionospheric channel model, which takes into account frequency dispersion, is proposed in [8]. We consider version of this model with a linear dispersion characteristic. Then frequency response of the ionospheric channel in the absence of multipath signal propagation can be described as

(2) $$\dot{H}(j2\pi f)=e^{-j\pi sf^{2}},\;f\in \left[-\Delta f/2;\;\Delta f/2\right],$$

where Δf is the bandwidth of the ionospheric channel.

The decision statistic can be found as:

(3) $${\dot{\lambda }}_{i}(\varphi ,\tau ,f_{d},s)=\sum_{n=0}^{N_{p}-1}{\dot{y}}_{n}(\varphi ,\tau =l\cdot \Delta t,f_{d},s){\dot{g}}_{i-n}^{*}(f_{d},s),$$

where the matched filter impulse response $\dot{g}$ is defined as

(4) $${\dot{g}}_{N_{p}-1-i}(f_{d},s)=\sum_{n=0}^{N_{p}-1}{\dot{x}}_{n}e^{j2\pi f_{d}n\Delta t}{\dot{h}}_{i-n}^{*}(s).$$

Then, the parameter estimates can be found as:

(5) $$\hat{\varphi },\hat{\tau },\hat{f_{d}},\hat{s}=\underset{\varphi ,\tau ,f_{d},s}{\arg\max}{\dot{\lambda }}_{i}(\varphi ,\tau ,f_{d},s),$$

where $\hat{\varphi },\hat{\tau },\hat{f_{d}}$ and $\hat{s}$ are estimates of $\varphi ,\tau ,f_{d}$ and s, respectively.

## 4. Implementation of a Matched Filter

This section may be divided by subheadings. It should provide a concise and precise description of the experimental results, their interpretation, and the experimental conclusions that can be drawn. From Equation (2) it can be seen that the number of matched filters to obtain a complete set of decision statistics ${\dot{\lambda }}_{i}(\varphi ,\tau ,f_{d},s)$ is determined by the number of possible Doppler frequency shifts $f_{d}$ and slopes of the dispersion characteristic s:

(6) $$N_{mf}=N_{f_{d}}N_{s},$$

where $N_{mf}$ is the number of matched filters, $N_{f_{d}}$ is the number of possible Doppler frequency shifts $f_{d}$, and $N_{s}$ is the number of possible slopes of the dispersion characteristic s. A large number of matched filters imposes high requirements on the computing platform. Doppler frequency shift $f_{d}$ consideration (for its estimation) can be carried out after matched filtering, then Equation (2) can be represented as:

(7) $${\dot{\lambda }}_{i}(\varphi ,\tau ,f_{d},s)=e^{j2\pi f_{d}i\Delta t}\sum_{n=0}^{N_{p}-1}{\dot{y}}_{n}(\varphi ,\tau =l\cdot \Delta t,f_{d},s){\dot{g}}_{i-n}(s),$$

where

(8) $${\dot{g}}_{N_{p}-1-i}(s)=\sum_{n=0}^{N_{p}-1}{\dot{x}}_{n}{\dot{h}}_{i-n}^{*}(s).$$

**Figure 1:**
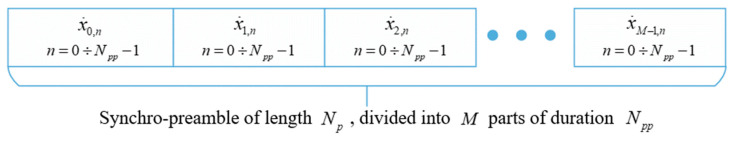
Reference signal divided into M parts.

The above transformation reduces number of required matched filters to $N_{mf}=N_{s}$, which can significantly reduce computational costs. However, in the conditions of an ionospheric channel, due to the presence of a Doppler frequency shift during the observation of the complex envelope at the input of the matched filter, a phase drift occurs, which leads to losses in the SNR at the output of the matched filter. To minimize these losses, we will convolve not with a reference signal of duration $N_{p}$, but with signals (see Figure 1):

(9) $${\dot{x}}_{m,n}={\dot{x}}_{n+m\cdot N_{pp}},\;n=0÷N_{pp}-1,\;m=0÷M-1,$$

where $N_{pp}=\frac{N_{p}}{M}$, and M is the number of splits of the original sequence.

In this case, matched filtering can be performed using a series-matched filter, which is a set of filters matched with sequences ${\dot{x}}_{m,n}$.

### 4.1. Estimation Algorithm via Complex Exponents

**Figure 2:**
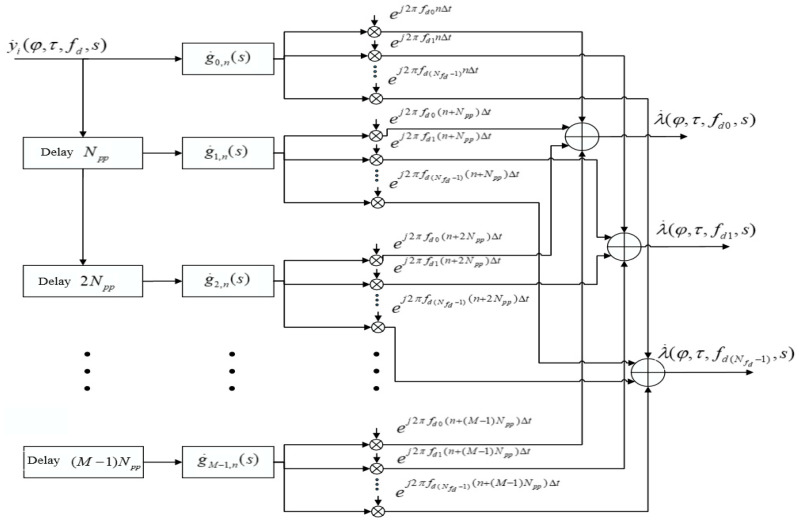
Matched filter with a series of sequences with complex exponents.

A filter matched with a series of sequences is shown at Figure 2. The signal at the output of each matched filter can be written as:

(10) $${\dot{\lambda }}_{m,n}(s)=\sum_{l=0}^{N_{pp}-1}{\dot{y}}_{m,l}{\dot{g}}_{m,n-l}^{*}(s),\;n=0÷N_{pp}-1,\;m=0÷M-1,$$

where ${\dot{g}}_{M-1-m,N_{pp}-1-n}(s)=\sum_{k=0}^{N_{p}-1}{\dot{x}}_{{}_{k+mN_{pp}}}{\dot{h}}_{n-(k+mN_{pp})}^{*}(s)$ is the complex impulse response envelope of the filter matched to the m-th sequence.

Doppler frequency shift is taken into account:

(11) $$\;{\dot{\lambda }}_{m,n}(f_{d},s)={\dot{\lambda }}_{m,n}(s)\cdot e^{j2\pi f_{d}(n+mN_{pp})\Delta t}.$$

The decision statistics at the matched filter output can be obtained as:

(12) $${\dot{\lambda }}_{n}(f_{d},s)=\sum_{m=0}^{M-1}{\dot{\lambda }}_{m,n}(f_{d},s).$$

The interval of allowable values of the Doppler frequency shift is $\left[-\frac{f_{s}}{2N_{pp}}:\frac{f_{s}}{2N_{pp}}\right]$, where $f_{s}$ is the sample rate. Within this interval, value of the estimated Doppler frequency shift can be arbitrary. A significant drawback of this implementation is the requirement for the amount of RAM to store arrays with complex exponents.

**Figure 3:**
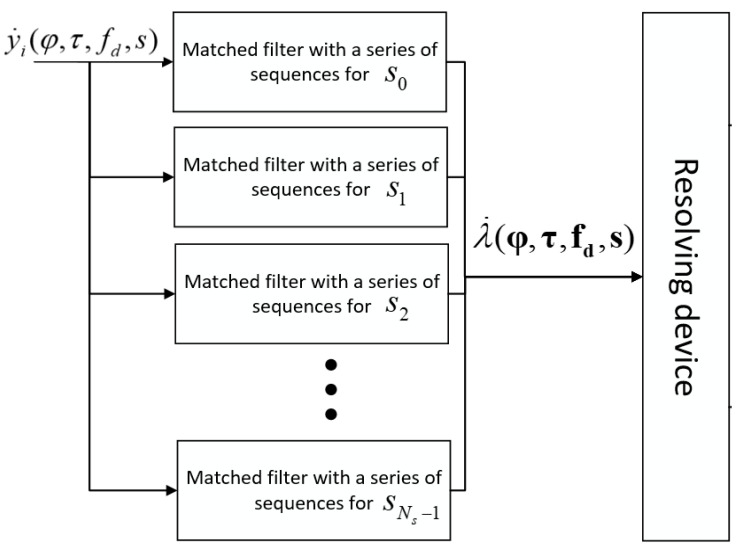
Scheme of the device for joint detection and signal parameter estimation.

Joint detection and signal parameters estimation device scheme is shown in Figure 3.

### 4.2. Algorithm with Doppler Estimation via FFT

Multiplication by complex exponents and the subsequent summation to further estimate the Doppler frequency shift can be done using the FFT.

Let $f_{d}=k\frac{f_{s}}{N}$, then Equation (10) can be represented as:

(13) $${\dot{\lambda }}_{n,k}(f_{d}=k\cdot \Delta f,s)=\sum_{m=0}^{M-1}{\dot{\lambda }}_{m,n}(s)\cdot e^{j2\pi km},$$

where

(14) $$\begin{array}{l} {\dot{\lambda }}_{m,n}(s)=\sum_{l=0}^{N_{pp}-1}{\dot{y}}_{m,l}{\dot{g}}_{m,n-l}^{*}(s),\\ n=0÷N_{pp}-1,m=0÷M-1. \end{array}$$

**Figure 4:**
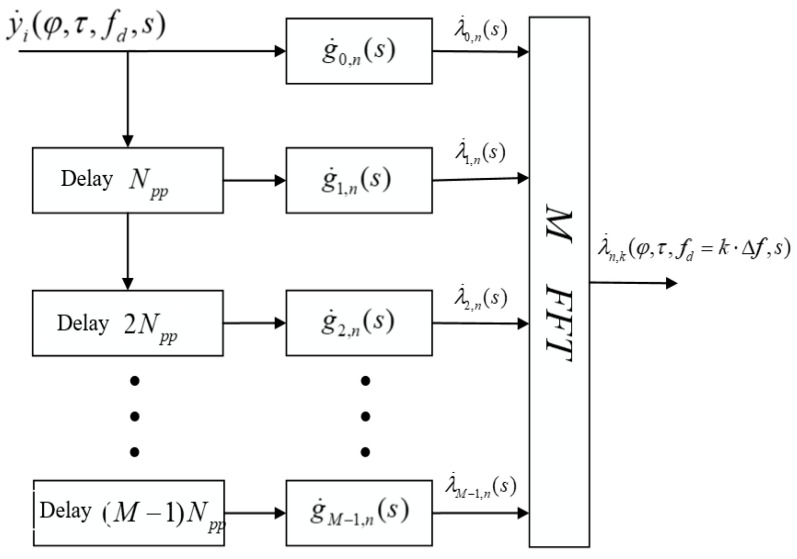
Matched filter with a series of sequences with searches over Doppler frequency shifts via FFT.

Equation (11) can be calculated using FFT algorithms from ${\dot{\lambda }}_{m,n}(s)$ for each k. This algorithm, in contrast to the algorithm with multiplications by complex exponents, makes it possible to estimate the Doppler frequency shift only for $f_{d}=k\cdot \Delta f$, where $k=[-\frac{N_{pp}}{2}:\frac{N_{pp}}{2}]$. The scheme of the filter matched with a series of sequences with searches for Doppler frequency shifts through the FFT is shown in Figure 4.

## 5. GPU Implementation

**Figure 5:**
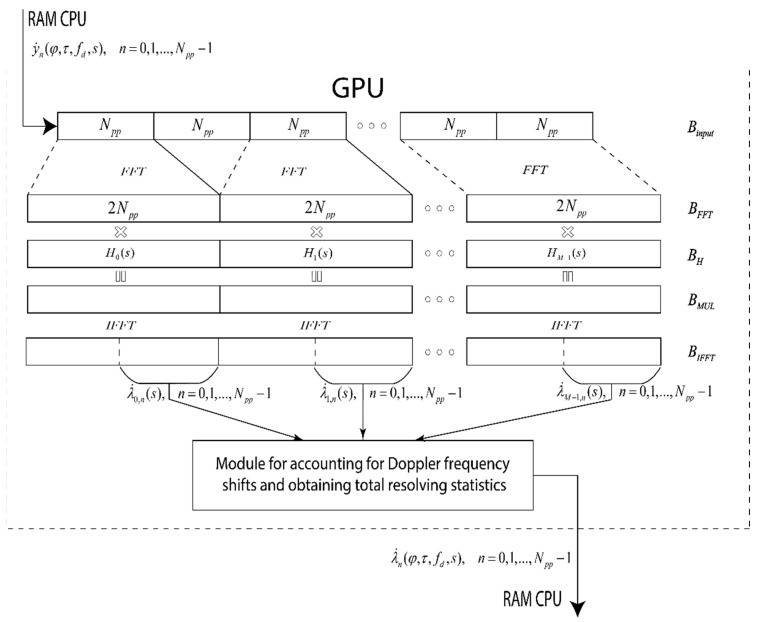
Implementation diagram of a matched filter with a series of sequences.

A matched filter with a series of sequences on the GPU is implemented using the fast convolution algorithm “Overlap and Save” [36] and the FFT and IFFT parallel computation library on the GPU–clFFT, implemented on OpenCL [37] (see Figure 5). The clFFT library is developed by clMathLibraries, an OpenCL library implementation of discrete fast Fourier transforms. The input data are loaded into the GPU in blocks of $N_{pp}$ samples. Loading is performed into a circular buffer $B_{input}$, size $N_{pp}(M+1)$. After loading the next block of samples, the buffer $B_{input}$ is fed to the calculation of the FFT with the size of $2N_{pp}$ with an overlap in $N_{pp}$ samples. FFT results are written to a buffer $B_{FFT}$, size $2N_{pp}M$. Post-FFT samples are multiplied with frequency response samples $\begin{array}{cc} H_{i}(s), & i=0,1,\ldots ,M-1 \end{array}$. The multiplication result is written to the buffer $B_{MUL}$ and fed to the calculation of the IFFT, size $2N_{pp}$. Samples after this IFFT are placed in the $B_{IFFT}$ buffer. The second half of each $2N_{pp}$ sample is the response of the filter ${\dot{\lambda }}_{m,n}(s)$ matched to the m-th sequence.

Received responses are transferred to the module for taking into account Doppler frequency shifts and obtaining the total decision statistics. This module is made in two versions. The first option is to directly multiply by complex exponents and then sum the filter responses. Multiplication operations by complex exponents are performed by calculating different samples of decision statistics using different GPU work items (WI).

**Figure 6:**
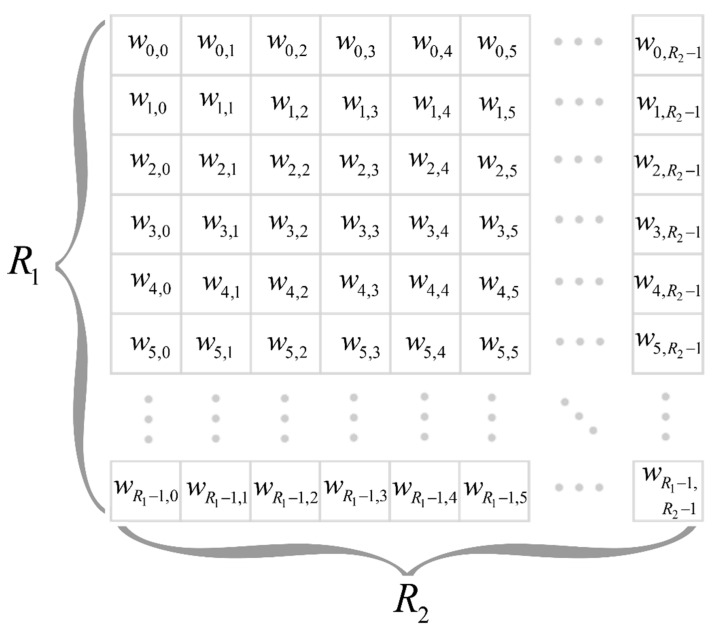
A set of GPU work items.

The work items set $w_{i,j}$ of the graphic processor is represented as a matrix W, dimension $R_{1}\times R_{2}$ (see Figure 6). Where $R_{1}$ and $R_{2}$ are numbers of work items in the 1st and 2nd dimension, respectively. These values determined by GPU implementation and have to be taken into account in the parallelization of the algorithm adaptation for GPU.

Within the available number of work items, it is proposed to parallelize the calculation of all samples of the decision statistics for all possible values of the Doppler frequency shifts $f_{d}$. The required number of work items to compute decision statistic samples ${\dot{\lambda }}_{n}(f_{d},s)$ for a single Doppler frequency shift value is $N_{pp}$. The maximum number of work items per calculation of the decision statistic samples for one value of the Doppler frequency shift can be calculated as:

(15) $$N_{\max\_items\_\exp}=\left\lfloor \frac{R_{1}R_{2}}{N_{f_{d}}}\right\rfloor .$$

Then, the actual number of work items is defined as:

(16) $$N_{items\_\exp}=\min(N_{\max\_items\_\exp},N_{pp}).$$

In the case when required number of work items exceeds number of available GPU items, some work items will calculate several samples of decision statistics ${\dot{\lambda }}_{n}(f_{d},s)$.

When performing calculations on the GPU, work items are combined into work groups (WG). The best performance is achieved by setting the work group size $N_{size\_work\_group}$ to the maximum, which is determined by the specific GPU implementation. The number of work groups for computing decision statistics samples ${\dot{\lambda }}_{n}(f_{d},s)$ for one value of Doppler frequency shift:

(17) $$N_{work\_group}=\left\lceil \frac{N_{items\_\exp}}{N_{size\_work\_group}}\right\rceil .$$

**Figure 7:**
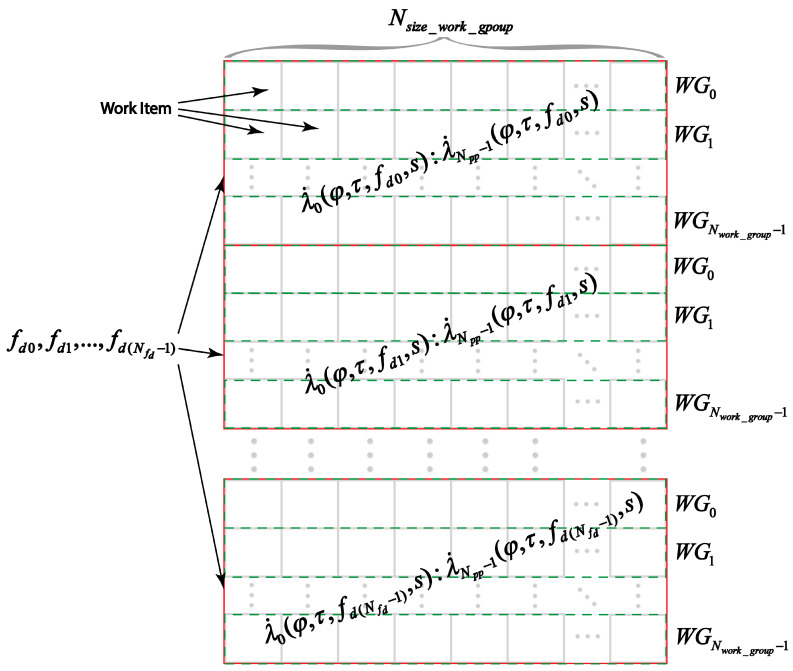
Distribution of computations between GPU work items.

The distribution of calculations between work items and GPU work groups is shown in Figure 7. This figure shows that the decision statistics values calculation ${\dot{\lambda }}_{n}(\varphi ,\tau f_{d},s)$ is divided into $N_{f_{d}}$ groups by $N_{work\_group}\times N_{size\_work\_group}$ work items. Each of these groups performs the calculation of the decision statistics samples ${\dot{\lambda }}_{n}(\varphi ,\tau f_{d},s)$ for one of the possible values of the Doppler frequency shift $f_{d}$. This improves the performance of the algorithm by performing parallel computations.

**Figure 8:**
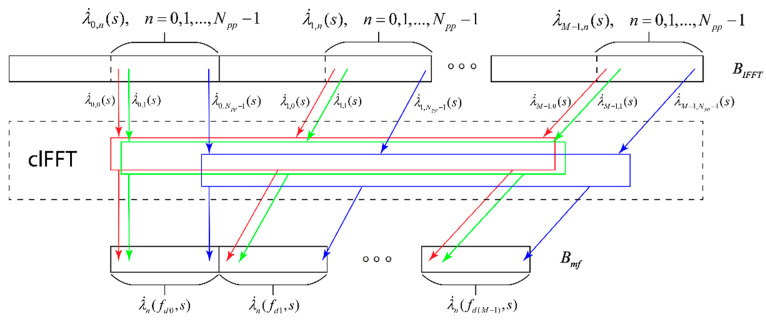
Scheme of the module for taking into account Doppler frequency shifts and obtaining the total decisive statistics, implemented through the FFT.

The second option for building a module for taking into account Doppler frequency shifts and obtaining the total decision statistics was performed using the FFT through the clFFT library. According to Equation (11) and Figure 4, the FFT must be taken from the n-th samples of all responses ${\dot{\lambda }}_{m,n}(s)$. The clFFT library allows you to perform all the necessary FFTs using a buffer $B_{IFFT}$ without additional memory operations. Figure 8 shows that the clFFT library allows you to perform an FFT from all n-th samples for all ${\dot{\lambda }}_{m,n}(s)$, $n=0÷N_{pp}-1,m=0÷M-1$ that were in the buffer $B_{IFFT}$ without additional data copies. The number of these FFT operations is M.

The FFT results are written to the buffer $B_{mf}$ in such a way that the decision statistics ${\dot{\lambda }}_{n}(f_{d},s)$ for different values of the Doppler frequency shift are sequentially stored in the memory.

## 6. Comparison of Algorithms Computational Complexity

Computational complexity is affected by the number of possible values $f_{d}$ and s, which are defined as $N_{s}$ and $N_{f_{d}}$, respectively. Computational complexity is given in the number of complex multiplications per one input sample. Computational complexity of the device for joint detection and estimation of signal parameters for two implementations of the algorithm is defined as:

(18) $$N_{cm}=\left(2M\left(\log_{2}(2N_{pp})-1\right)+MN_{f_{d}}\right)N_{s},$$

(19) $$N_{cm\_fft}=\left(2M\left(\log_{2}(2N_{pp})-1\right)+\frac{N_{f_{d}}}{2}\left(\log_{2}(N_{f_{d}})-2\right)\right)N_{s}.$$

Thus, computational complexity of the proposed algorithm depends on the number of partitions of the original sequence M, the duration of one part of the original sequence $N_{pp}$, the number of possible values of Doppler shifts in frequency $N_{f_{d}}$, and slopes of the dispersion characteristic of the ionospheric channel $N_{s}$.

## 7. Test Results on CPU and GPU

**Figure 9:**
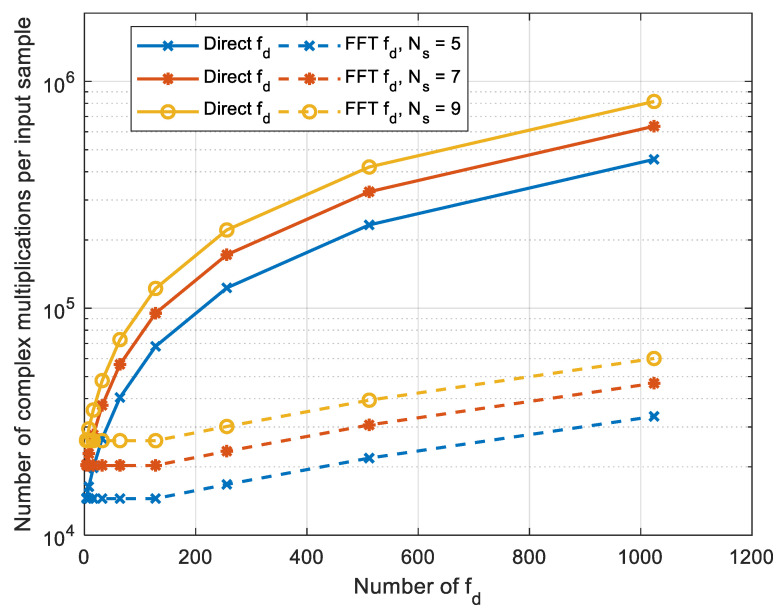
Dependence of the number of complex multiplications on the number of possible values $f_{d}$ for a different number of possible values of the slope of the dispersion characteristic of the ionospheric channel $N_{s}$, M=86, $N_{pp}=32768$.

For the experiment, a six-core Intel Core i7-8700 CPU with a clock frequency of 3.2 GHz and a Geforce RTX 3060 GPU with 3584 CUDA cores, a base clock frequency of 1.32 GHz, and a 192-bit memory bus were used. The experiment was run on a computing platform of 32 GB of RAM with a speed of 2400 MT/s. The experiment was carried out in the operating system Linux Ubuntu 20.04 with Nvidia GPU driver version 460.73.01. The used clFFT library version was 2.12.2. For algorithm implementation, compilation was used with a gcc 9.4.0 compiler with compiler flags set to o2. To execute calculations, five cores and 10 threads of Intel Core i7-8700 CPU were used. One core and two threads were left for the needs of the operating system. Testing was performed on a signal with a bandwidth ΔF=400 kHz and a duration T=7 s. The base of this signal was 64.5 dB. These parameters were chosen based on the results of field experiments carried out on single-hop ionospheric paths up to 3000 km long. The search ranges for the Doppler frequency shift and the slope of the dispersion characteristic of the ionospheric channel were also selected based on the results of field experiments. Dependence of the computational complexity on the number of possible values of Doppler shifts in frequency $N_{f_{d}}$ for a different number of slopes of the dispersion characteristic of the ionospheric channel $N_{s}$ for $N_{pp}=32768$ and M=86 is shown in Figure 9.

**Figure 10:**
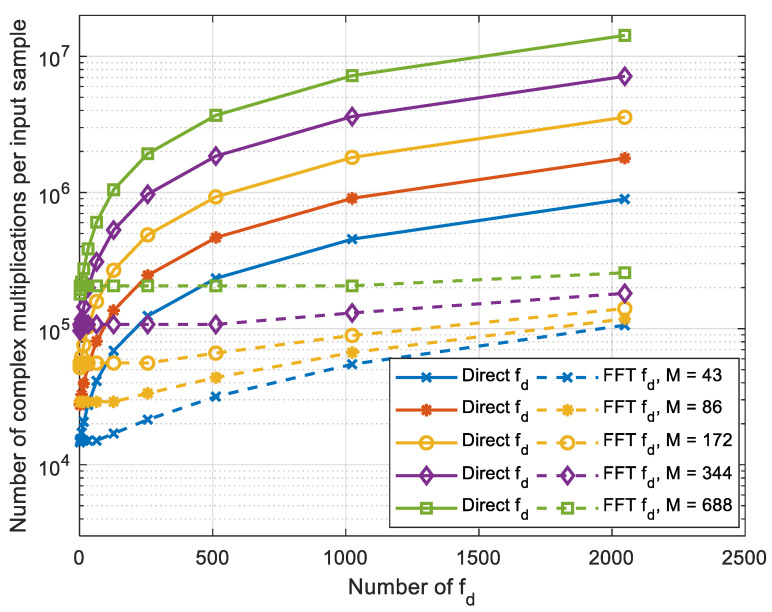
Dependence of the number of complex multiplications on the number of possible values $f_{d}$ for a different number of splits M of the original signal at $N_{s}=10$.

This graph shows that an increase in the number of possible values $N_{f_{d}}$ leads to a slight increase in computational complexity compared to an increase in the number of possible values $N_{s}$. The dependence of the number of complex multiplications on the number of possible values $f_{d}$ for a different number of splits M of the original signal at is shown in Figure 10.

The number of experiments performed to obtain averaged results was 1000. Increasing the number M leads to an increase in computational complexity.

Table 1 shows the dependence of the algorithm running time on the block duration for $\begin{array}{cc} f_{d}=-5:0.05:5 & N_{f_{d}}=201 \end{array}$.

Table 2 shows how many times RTX 3060 GPU is faster than base Intel i7-8700 processor. It can be seen that the performance gain of the RTX 3060 GPU in the algorithm without FFT decreases with increasing block duration, while in the algorithm with FFT, it remains constant.

The TDP of the RTX 3060 GPU is 170W, while the TDP of the Intel Core i7-8700 is 65W. Thus, the increase in power consumption when using the RTX 3060 GPU compared to the Intel Core i7-8700 CPU is 2.62 times, and the minimum performance increase is 4.37 times. Therefore, it is advisable to use a GPU, since the increase in performance exceeds the loss in power consumption.

**Figure 11:**
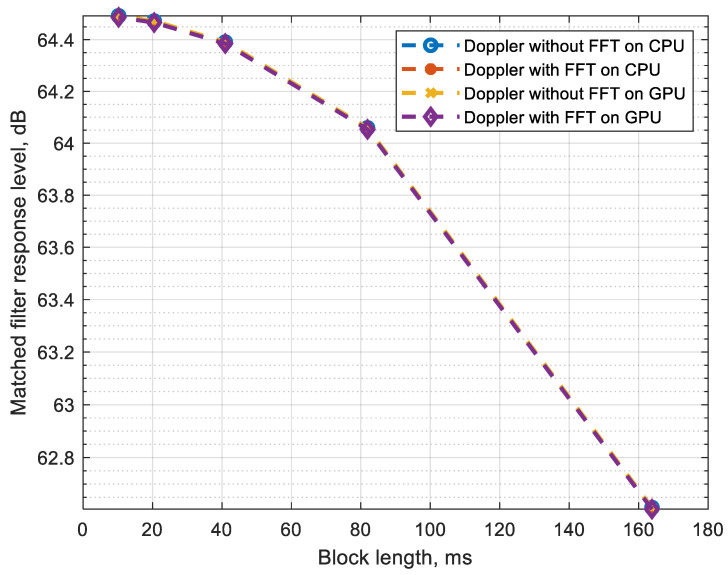
Dependence of the response level of the matched filter on the duration of the block with a Doppler frequency shift $f_{d}=3$.

Dependence of the response level of the matched filter on the block duration at the Doppler shift $f_{d}=3$ is shown in Figure 11.

Implementation with Doppler shift estimation via FFT on the GPU is the most efficient and allows for processing one sample in less than 2 µs with a loss of no more than 0.5 dB. With a block duration of less than 80 ms, the loss does not exceed 0.5 dB.

## 8. Conclusions

This paper proposes two implementations of the joint detection and estimation of the parameters of signals with dispersion distortions on the CPU and GPU. In the first method, the estimation of the Doppler frequency shift is performed in a direct way, by multiplying by complex exponents. In the second method, estimation of the Doppler frequency shift is performed through the FFT. All FFTs in the proposed implementations are performed through the “Overlap and Save” fast convolution algorithm. The computational complexity of the proposed implementations of joint detection and estimation of signal parameters is calculated. It is shown that the method based on the estimation of the Doppler frequency shift through the FFT is the most computationally efficient. Implementation of this method on the GPU allows for the joint detection and estimation of signal parameters in real time. It is shown how the duration of a block in a matched filter with a series of sequences affects the response level. Reducing the block duration results in a reduction in matched response level loss but results in an increase in computational complexity.

## Figures and Tables

**Table 1:** Experimental running time of the algorithms per one input sample, with different block durations.

Algorithm Implementation Type	Block Length 10.24 ms µs	Block Length 20.48 ms µs	Block Length 40.96 ms µs	Block Length 81.92 ms µs	Block Length 163.84 ms µs
Doppler without FFT on CPU	251.1	124.4	62.59	31.3	15.91
Doppler with FFT on CPU	17.83	9.17	5.88	3.98	2.51
Doppler without FFT on GPU	7.36	4.21	2.49	1.61	1.19
Doppler with FFT on GPU	3.91	2.03	1.29	0.91	0.55

**Table 2:** GPU RTX 3060 Performance Boost vs. CPU Intel Core i7-8700.

Algorithm Implementation Type	Block Length 10.24 ms	Block Length 20.48 ms	Block Length 40.96 ms	Block Length 81.92 ms	Block Length 163.84 ms
Doppler without FFT	34.12	29.55	25.14	19.44	13.37
Doppler with FFT	4.56	4.52	4.56	4.37	4.56

## Data Availability

Not applicable.
